# Schistosomiasis in Africa: Improving strategies for long-term and sustainable morbidity control

**DOI:** 10.1371/journal.pntd.0006484

**Published:** 2018-06-28

**Authors:** Michael D. French, Darin Evans, Fiona M. Fleming, W. Evan Secor, Nana-Kwadwo Biritwum, Simon J. Brooker, Amaya Bustinduy, Anouk Gouvras, Narcis Kabatereine, Charles H. King, Maria Rebollo Polo, Jutta Reinhard-Rupp, David Rollinson, Louis-Albert Tchuem Tchuenté, Jürg Utzinger, Johannes Waltz, Yaobi Zhang

**Affiliations:** 1 RTI International, Washington DC, United States of America; 2 United States Agency for International Development, Washington, DC, United States of America; 3 Schistosomiasis Control Initiative, Imperial College London, London, United Kingdom; 4 Centers of Disease Control and Prevention, Atlanta, Georgia, United States of America; 5 Bill and Melinda Gates Foundation, Seattle, Washington, United States of America; 6 London School of Hygiene and Tropical Medicine, London, United Kingdom; 7 Global Schistosomiasis Alliance, London, United Kingdom; 8 Schistosomiasis Control Initiative, Kampala, Uganda; 9 Case Western Reserve University, Cleveland, Ohio, United States of America; 10 Expanded Special Program for Elimination of NTDs (ESPEN), World Health Organization Regional Office for Africa, Brazzaville, Republic of Congo; 11 Global Health Institute, Merck KGaA (Germany), Coinsins, Switzerland; 12 University of Yaoundé I, Yaoundé, Cameroon; 13 Centre for Schistosomiasis and Parasitology, Yaoundé, Cameroon; 14 Swiss Tropical and Public Health Institute, Basel, Switzerland; 15 University of Basel, Basel, Switzerland; 16 Helen Keller International, Dakar, Senegal; Saudi Ministry of Health, SAUDI ARABIA

## Background

Schistosomiasis affects over 200 million people worldwide [1] and accounts for an estimated 1.9 million disability-adjusted life years (DALYs) annually [2], with 90% of the burden currently concentrated in Africa. The last decade has witnessed an extraordinary surge of advocacy and funding for neglected tropical diseases (NTDs), including schistosomiasis. Large-scale schistosomiasis control is now implemented in 30 countries in Africa [1], funded primarily through support from the United States Agency for International Development (USAID) and the Department for International Development (DFID), private philanthropic funds from the END Fund and through GiveWell recommendations, and leveraging praziquantel donations from Merck KGaA. However, the number of people still requiring treatment remains daunting [1].

**Fig 1:**
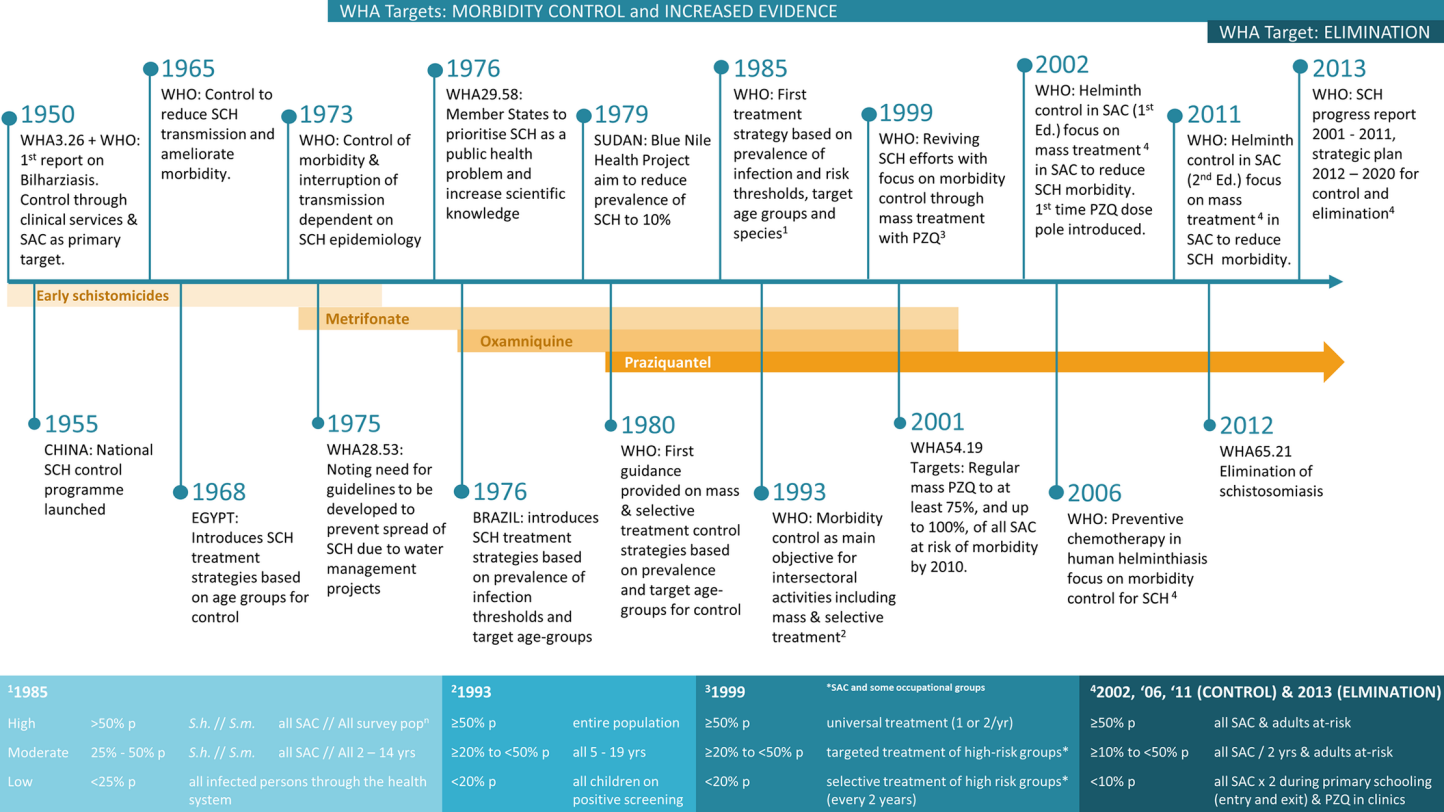
Timeline of global schistosomiasis control and elimination strategies (1950–present). p, prevalence; PZQ, praziquantel; SAC, school-age children; S.h., *Schistosoma haematobium*; S.m., *Schistosoma mansoni*; SCH, schistosomiasis; WHA, World Health Assembly; WHO, World Health Organization; yrs, years.

The aim of current public health strategies for schistosomiasis is to decrease morbidity through preventive chemotherapy (PC) (Fig 1) [3]. Periodic large-scale administration of the drug praziquantel focusing on the school-aged population and high-risk adults aims to reduce the prevalence and intensity of infection [4].

## Progress on elimination of transmission

The exciting progress towards elimination of other NTDs with targeted end dates as embodied in the London Declaration of 2012 and the World Health Organization (WHO)’s 2020 Roadmap [5,6] has led to a similar push to interrupt transmission of schistosomiasis. This culminated in the 2012 World Health Assembly resolution 65.21 calling on countries to intensify control and initiate elimination campaigns “where appropriate” [7]. While enthusiasm for this goal is understandable, too often, the resolution’s modifier of “where appropriate” is overlooked, and it is forgotten that places where elimination efforts have been successful were either isolated or had sustained environmental or major economic changes, usually through improved sanitation and aggressive economic development [8,9]. By contrast, most of sub-Saharan Africa relies on PC as the only control intervention and often within a restricted age group (typically school-aged children). In the absence of significant socioeconomic development, elimination of transmission remains an elusive, or at least distant, goal in most countries.

## Refocus on morbidity control

Treatment guidelines have changed little since their initial development two decades ago [10]. They stem from an era when drug supplies were costly and limited and infection-associated morbidities less well understood.

Since then, the schistosomiasis field has progressed. Praziquantel is now donated in large amounts by Merck KGaA, and knowledge of schistosomiasis morbidity, while still imperfect, is stronger. Schistosomiasis morbidity is broad and encompasses acute (e.g., anemia, diarrhea, and pain), chronic (e.g., portal hypertension, hepatic fibrosis, and cognitive impairment), anatomical (e.g., hepatomegaly and intestinal hemorrhage), functional (e.g., exercise intolerance, poor school performance, and loss of earnings [11]), and even post-infection morbidity (male and female genital schistosomiasis and increased HIV transmission risk [12]). However, the relationship between infection and morbidity is complex. Age of first infection, infection intensity and duration, parasite species, and coinfections affect the types of morbidity experienced. Many of these are multifactorial, complicating their use as potential schistosomiasis morbidity markers. Recent evidence suggests that morbidity extends beyond just school-aged children [13], but school-aged children remain the focus of control programs.

It is timely to refocus schistosomiasis programs (both intestinal and urogenital, caused by *Schistosoma mansoni* and *S*. *haematobium*, respectively) in Africa on improving strategies for long-term and sustainable morbidity control until a decision to pursue elimination can be made. With realistic, evidence-based targets, programs can more readily measure progress and adapt approaches. Similarly, drug donors and funders can ensure their contributions are having measurable impact, which is crucial to be able to justify continued support.

To develop guidelines based on morbidity control, we envisage a framework developed around three crucial questions.

### How is morbidity control defined?

The current morbidity control program indicators for intestinal and urogenital schistosomiasis of 1% and 5% heavy infection [3] came from the mantra that “risk of morbidity is due to heavy infection,” which is based on expert opinion in the 1980s when praziquantel was not widely available, diagnostics relied on egg identification, and morbidity was defined as severe fibrotic manifestations of chronic infection. We now have an opportunity to update these targets given the new understanding of morbidity and more sensitive diagnostics. The ideal definition of morbidity control would be the identification of a level of infection below which little to no morbidity occurs and a marker that is readily measurable in the field as part of large-scale control programs. To identify such markers will require a deeper understanding of the link between infection and morbidity and how this varies in different populations (such as preschool-aged children and adults) at different stages of control (such as pre- and post-treatment), and whether morbidity in one group (e.g., school-aged children) is a useful proxy for other population groups. This will require a synthesis of already-available data, operational research to fill evidence gaps, and modeling studies. The output will hopefully be species-specific (intestinal and urogenital) morbidity targets around which the schistosomiasis community can coalesce. If areas are endemic for both *S*. *mansoni* and *S*. *haematobium*, it may be necessary to consider whether coinfection results in exacerbated morbidity over each infection alone. In Table 1, we provide a non-exhaustive list of potential schistosomiasis-related proxy morbidity markers.

**Table 1:** Potential morbidity metrics for urogenital and intestinal schistosomiasis control programs in Africa.

Category of morbidity indicator	Urogenital schistosomiasis (*S*. *haematobium*)	Intestinal schistosomiasis (*S*. *mansoni*)
**Currently recommended primary measures [3]**	Prevalence of heavy infection (≥50 eggs/10ml) measured via urine filtration	Prevalence of heavy infection (≥400 eggs per gram of stool) via Kato–Katz thick smear testing
**Available alternatives:**		
Point-of-care test prevalences	Micro- and macrohematuria (blood in the urine)	Blood in the stool (including persistent bloody diarrhea)
	Proteinuria	Fecal occult blood
	Leukocyturia	Calprotectin in stool
	Anemia	Anemia
Prevalence of chronic and/or anatomic findings	Ultrasonography of bladder and ureters and genital organs	Ultrasonography of liver, spleen, portal branch, portal veins
	Palpation of bladder tenderness	Palpation of liver and spleen size
	FGS signs and symptoms score (vaginal discharge, bleeding after intercourse, genital itching, pelvic pain)	Ascites
	MGS signs and symptoms score (hemospermia, egg excretion in semen, prostatic enlargement)	
	Growth stunting (height for age)	Growth stunting (height for age)
	Abnormally low BMI (physical wasting)	Abnormally low BMI (physical wasting)
Quantifiable functional morbidities among SAC	Shuttle run test for exercise intolerance	Shuttle run test for exercise intolerance
	School attendance and behavior	School attendance and behavior
	Cognitive development	Cognitive development

**Abbreviations:** BMI, body mass index; FGS, female genital schistosomiasis; MGS, male genital schistosomiasis; SAC, school-aged children.

We contend that redefining morbidity control targets is a priority for the schistosomiasis community and will allow the subsequent answering of how to achieve and demonstrate morbidity control.

### How is morbidity control achieved?

Once a control target is identified, it then becomes possible to define the control strategy required to achieve it.

School-aged children have traditionally been the focus of both treatment and epidemiologic evaluation because of their high risk of infection. Infection and morbidity risk, however, extends to preschool-aged children, women of reproductive age, and other high-risk groups (e.g., car washers, fishermen, and rice farmers). Furthermore, while the correlation between infection and morbidity within age groups is strong, the correlation between age groups is weaker [14], suggesting that treatment of additional at-risk groups may be required to achieve comprehensive morbidity control.

There is a strong argument for treating preschool-aged children to prevent morbidity before it develops [13]. At present, however, preschoolers are not included in PC programs because of the challenge of treating small children with large, bitter-tasting pills. Pediatric praziquantel is projected to be available within 2 years [13], although not on a mass-treatment platform. It will be necessary to define which preschool-aged children in which areas should be treated and at what interval. Similarly, guidance is needed on which adults should receive treatment, how frequently, and for how long. Strategies for identifying and treating foci of intense schistosomiasis transmission despite good PC coverage (hot spots) must also be developed to ensure the optimum use of scarce resources. In all age groups, targets should be aligned with the ultimate goal of the programs and therefore measured in terms of prevalence of infection or heavy infection using sensitive tools. To consolidate gains in PC, it may also be necessary to consider the dynamics of environmental transmission in the local aquatic habitat and implement synergistic intervention measures [15].

### How is morbidity control demonstrated?

It will not be straightforward to demonstrate that morbidity has been controlled. The adoption of robust and standardized monitoring and evaluation framework describing the sampling strategy, the required frequency of surveys, and the populations to be tested are required. The choice of populations in which assessments should be conducted (e.g., school-aged children only, adults, or entire communities) will be affected by the morbidity control target and strategies to achieve it. The choice of the infection target will need to incorporate the imperfect sensitivity of the diagnostic tools: Kato–Katz and urine filtration, for example, are insensitive, particularly at low levels of infection, and may correlate poorly with morbidity. Additionally, egg-negative morbidity is possible, such as in patients with female genital schistosomiasis [16]. The addition of tools such as the point-of-care circulating cathodic antigen (POC-CCA; field ready) and the circulating anodic antigen (CAA; under development) will help in this regard but need to be evaluated in relation to morbidity.

It will also be important to determine if, once morbidity control is achieved, intervention efforts can be backed off to maintain program gains. While continuing PC indefinitely is not attractive to programs, donors, or recipients, public health programs such as vitamin A, school health days, and immunization campaigns provide models for effective sustained interventions.

## Call for action

To summarize, we see the key points for developing valuable new schistosomiasis morbidity control guidelines on the following grounds:

- Schistosomiasis morbidity remains a major public health problem in sub-Saharan Africa.
- Interruption of transmission at a national scale remains the ultimate objective, but this requires more than PC and is a long-term endeavor in most countries.
- In the meantime, greater attention is needed to address how morbidity control can be defined, achieved, and demonstrated.
- Available scientific research on schistosomiasis-related morbidity has increased in recent years, but there remain knowledge gaps on the link between infection and morbidity.
- There is a recognized need to update the current WHO guidelines for morbidity control.
- Morbidity control targets should be realistic and measurable.
- Utilization of valuable drug and implementation resources needs to be as effective as possible.

Evidence gathered over the past decade can improve the impact of PC strategies for morbidity control. Additionally, we recommend efforts to develop a core operational research agenda to identify priority knowledge gaps to help improve the impact of programs.

## Conclusion

Considerable financial and human resources and long-term political commitment will likely be needed to achieve elimination of schistosomiasis transmission in Africa. The current mainstay of schistosomiasis programs, PC, will need to be complemented by other control interventions such as increased access to clean water, improved sanitation and hygiene, communications to support behavior change, environmentally friendly snail control, and a vaccine. These additional elements remain beyond the scope of realistic interventions and funding sources for most of sub-Saharan Africa. Therefore, while elimination of transmission rightly remains the aspirational, long-term goal, we recommend taking this opportunity to refocus on strategic investments in long-term morbidity control. We recommend that evidence-based schistosomiasis control program targets be developed that maximize resources and limit the morbidity caused by infection until country-specific shifts to elimination become feasible.

The continued leadership of WHO with support by the Global Schistosomiasis Alliance (GSA; http://eliminateschisto.org/) and the wider schistosomiasis research and implementation community will be crucial in this process.
